# Scrub typhus in Indonesia: A cross-sectional analysis of archived fever studies samples

**DOI:** 10.1093/trstmh/trad094

**Published:** 2024-01-11

**Authors:** Kartika Saraswati, Ampai Tanganuchitcharnchai, Sirada Ongchaikupt, Mavuto Mukaka, Nicholas P J Day, J Kevin Baird, Ungke Antonjaya, Khin S A Myint, Yora P Dewi, Frilasita A Yudhaputri, Sotianingsih Haryanto, N P Diah Witari, Stuart D Blacksell

**Affiliations:** Oxford University Clinical Research Unit Indonesia, Faculty of Medicine, Universitas Indonesia, 10430 Jakarta, Indonesia; Mahidol Oxford Tropical Medicine Research Unit, Faculty of Tropical Medicine, Mahidol University, 10400 Bangkok, Thailand; Centre for Tropical Medicine and Global Health, Nuffield Department of Medicine, University of Oxford, OX3 7LG Oxford, UK; Mahidol Oxford Tropical Medicine Research Unit, Faculty of Tropical Medicine, Mahidol University, 10400 Bangkok, Thailand; Mahidol Oxford Tropical Medicine Research Unit, Faculty of Tropical Medicine, Mahidol University, 10400 Bangkok, Thailand; Mahidol Oxford Tropical Medicine Research Unit, Faculty of Tropical Medicine, Mahidol University, 10400 Bangkok, Thailand; Centre for Tropical Medicine and Global Health, Nuffield Department of Medicine, University of Oxford, OX3 7LG Oxford, UK; Mahidol Oxford Tropical Medicine Research Unit, Faculty of Tropical Medicine, Mahidol University, 10400 Bangkok, Thailand; Centre for Tropical Medicine and Global Health, Nuffield Department of Medicine, University of Oxford, OX3 7LG Oxford, UK; Oxford University Clinical Research Unit Indonesia, Faculty of Medicine, Universitas Indonesia, 10430 Jakarta, Indonesia; Centre for Tropical Medicine and Global Health, Nuffield Department of Medicine, University of Oxford, OX3 7LG Oxford, UK; Oxford University Clinical Research Unit Indonesia, Faculty of Medicine, Universitas Indonesia, 10430 Jakarta, Indonesia; Exeins Health Initiative, 12870 Jakarta, Indonesia; Exeins Health Initiative, 12870 Jakarta, Indonesia; Exeins Health Initiative, 12870 Jakarta, Indonesia; Raden Mattaher Hospital, 36122 Jambi, Indonesia; Faculty of Medicine and Health Sciences, Universitas Jambi, 36361 Jambi, Indonesia; Faculty of Medicine and Health Sciences, Warmadewa University, 80235 Denpasar, Bali, Indonesia; Mahidol Oxford Tropical Medicine Research Unit, Faculty of Tropical Medicine, Mahidol University, 10400 Bangkok, Thailand; Centre for Tropical Medicine and Global Health, Nuffield Department of Medicine, University of Oxford, OX3 7LG Oxford, UK

**Keywords:** scrub typhus, *Orientia tsutsugamushi*, serology, Indonesia

## Abstract

**Background:**

Scrub typhus is an understudied vector-borne bacterial infection.

**Methods:**

We tested archived fever samples for scrub typhus seropositivity to begin charting its geographic distribution in Indonesia. We analysed 1033 serum samples from three sites. IgM and IgG enzyme-linked immunosorbent assay (ELISA) against *Orientia tsutsugamushi* was performed using Karp, Kato, Gilliam, TA 716 antigens. To determine the cutoff in the absence of a presumed unexposed population and gold standard tests, we identified the visual inflection point, performed change point analysis, and used finite mixture models.

**Results:**

The optical density cutoff values used for IgM and IgG were 0.49 and 0.13, respectively. Across all sites, IgM seropositivity was 4.6% (95% CI: 3.4 to 6.0%) while IgG seropositivity was 4.4% (95% CI: 3.3 to 5.8%). The overall seropositivity across sites was 8.8% (95% CI: 8.1 to 11.7%). The overall seropositivity for Jambi, Denpasar, Tabanan were 9.7% (95% CI: 7.0 to 13.3%), 8.0% (95% CI: 5.7 to 11.0%), 9.0% (95% CI: 6.1 to 13.0%), respectively.

**Conclusions:**

We conclude that *O. tsutsugamushi* exposure in humans occurred at all sites analysed and could be the cause of illness in some cases. Though it was not the main cause of acute fever in these locations, it is still important to consider scrub typhus in cases not responding to beta-lactam antibiotics. Future seroprevalence surveys and testing for scrub typhus in acute febrile illness studies will be essential to understand its distribution and burden in Indonesia.

## Introduction

Scrub typhus, a febrile illness transmitted to humans by mites, is the leading vector-borne bacterial infection in South and Southeast Asia.^1^ It is estimated that there are about one million cases annually with one billion people at risk.^2^ Scrub typhus is caused by bacteria of the genus *Orientia*. The primary aetiologic agent, particularly in the Asia Pacific region, is the bacterium *Orientia tsutsugamushi*. Recently, other candidate species have been described in Dubai and Chile: *Candidatus* Orientia chuto and *Candidatus* Orientia chiloensis, respectively.^3,4^ Those findings demonstrated that scrub typhus is not confined within the ‘tsutsugamushi triangle’—the area bordered by Pakistan and Afghanistan in the west, Northeast Russia and Japan in the northeast, and North Australia in the south—which is often described as its classical distribution area.^2^

Scrub typhus often presents as undifferentiated acute fever; therefore, laboratory diagnosis plays a central role.^2,5^ In Indonesia, scrub typhus diagnostics are very infrequently performed. Apart from a few referral laboratories or in research settings, laboratory tests to diagnose scrub typhus are rarely available, as are scrub typhus treatment guidelines. Many clinicians are unaware of scrub typhus and this lack of awareness combined with the scarcity of access to confirmatory diagnosis largely explains the meagre evidence regarding scrub typhus distribution in Indonesia.

Although scrub typhus active transmission and cases have been confirmed in Indonesia,^6–8^ the burdens of morbidity and mortality, geographic distribution, frequency or magnitude are almost wholly unknown. This blind spot obviously imposes the risk of dangerous underestimation of harm.

In this study we performed a serosurvey of scrub typhus using archived sera taken from patients seeking care for acute febrile illness to begin characterizing the geographic distribution and frequency of scrub typhus exposure in Indonesia.

## Materials and methods

This was a cross-sectional study of archived sera of acutely febrile participants. This study was approved by the Eijkman Institute Research Ethics Committee (approval No. 128) and received a waiver from the Oxford Tropical Research Ethics Committee (OxTREC) as the project analysed anonymized samples obtained in a clinical study previously cleared.

### Serum samples collection

We obtained and tested 1035 sera collected from 2014 to 2018 (Supplementary Data 1, Supplementary Table S1).^9,10^ The sera were collected as a part of acute febrile illness (AFI) studies across three hospitals. The datasets collected previously included demographic variables, clinical manifestations and laboratory values. These samples were previously tested for dengue infection with flavivirus PCR and dengue NS1.^9,10^ The sera were stored in −80°C freezers. We included all samples with minimum volume of 100 μl to ensure sufficiency and validity.

### Study size calculation

The minimum number of sera to be analysed per site was set at 200. If there were no seropositive samples and the sample size is 200, the upper limit of 95% CI—based on binomial distribution, would be 0.018, and thus we can be 95% certain that the true seropositivity in the population is less than 2%.

### Laboratory procedures

Indirect IgM and IgG enzyme-linked immunosorbent assay (ELISA) against *O. tsutsugamushi* was performed using the methodology previously described by Phanichrivalkosil et al.^11^ Briefly, half of the wells in the 96-well microtiter plates were coated with 100 μl of *O. tsutsugamushi* antigen from four strains (Karp, Kato, Gilliam, TA 716); while the other half were not coated. The plates were stored in a moist chamber at 4°C for 36–48 h. Two hundred microlitres of blocking solution (0.1% Tween 20 in 10X phosphate buffered saline [PBST] and 5% skimmed milk) was pipetted into each well and the plates were incubated in a moist chamber at room temperature for one hour. Then, the plates were washed three times using PBST as the wash buffer. Ten microlitres of participant sera were diluted in blocking buffer to make 1:100 dilution. We added 100 μl of diluted participant sera in one well with and one well without antigen coating. We used 5% skimmed milk as a negative control. Previously characterized participant sera were used as positive controls. After tapping gently to mix, the plates were then incubated for one hour in a moist chamber at room temperature and washed four times. Goat anti-human IgG (gamma chain) cross-adsorbed secondary antibody, horseradish peroxidase (HRP) conjugate (Invitrogen Corporation, Thermo Fisher Scientific, USA, catalogue number 62–8420) and goat anti-human IgM secondary antibody, HRP conjugate (Invitrogen Corporation, Thermo Fisher Scientific, USA, catalogue number 31 415) were used. For IgM and IgG conjugated antibody, we used 1:8 000 and 1:1 000 dilution, respectively. We added 100 μl conjugated antibody in each well, then incubated the plates for one hour in a moist chamber at room temperature and washed four times. One hundred microlitres of tetramethylbenzidine (TMB) substrate chromogen solution (Invitrogen Corporation, Thermo Fisher Scientific, USA, catalogue number 002 023) per well was added. The plates were incubated for 15 min in the dark at room temperature and then 100 μl 1M HCl stop solution was added.

The resulting optical density readings at 450 nm were subtracted from the readings at 620 nm (Thermo Scientific™ Multiskan™ FC, Singapore). Then, we subtracted the optical density of wells coated with antigen with wells without antigen to eliminate background/non-specific absorbance.

### Data analysis

Continuous variables were summarized in medians (Q1–Q3). Categorical variables were summarized in proportion and frequencies. The association between categorical (site, sex, dengue positive status) and continuous variables (age) with the scrub typhus seropositivity was analysed using the Chi-squared test and logistic regression, respectively. p-Values of less than 0.05 were considered statistically significant. Analysis was done with Microsoft Excel 2019 (Microsoft Corporation, Redmond, WA, USA), R version 3.6.2 (R Foundation for Statistical Computing, Vienna, Austria), R Studio Version 1.2.5033 (RStudio, Inc., Boston, MA, USA), and StataBE 17 (StataCorp, College Station, TX, USA). The reporting of this study followed the Strengthening the Reporting of Observational Studies in Epidemiology (STROBE) Statement (Supplementary Data 1, Supplementary Table S2). Samples with missing data were excluded from analysis if they were not compromising the required sample size number.

### Cutoff selection

The mean plus two or three standard deviations of the optical density values of the ‘presumed unexposed’ or ‘negative control’ samples were usually used as the cutoff to determine seropositivity.^12^ However, due to limitations imposed by the Covid-19 pandemic, we could not test samples of this population. There were no established scrub typhus ELISA cutoffs in the sites analysed, or Indonesia in general. We used finite mixture models to determine the cutoff since we deemed this method to be more objective. We also identified the visual inflection point and performed change point analysis as comparisons.

#### Finite mixture model

Populations often consist of two or more subpopulations.^12,13^ These subpopulations are defined by certain characteristics such as age brackets, sex, economic status, or education levels.^13^ However, there are cases when the identifying variable is difficult to collect or unobservable.^13,14^ Finite mixture models (FMMs) can model the probability of each observation fitting in each subpopulation.^13,14^ They can classify samples based on disease status when clinical measurements or laboratory parameter values are available, but ‘individual class memberships are not available’ or too difficult or expensive to gather.^14^

In the context of our study, this approach would be able to categorize the data into two groups: the seronegative and seropositive groups. The seronegative group would have lower optical density values.^12^ Thus, if there are two groups with normal distribution, the lower distribution would belong to the seronegative population. The mean and variance of the lower class were used to calculate the cutoff, i.e. the mean plus three standard deviations (mean+3SD) of the lower distribution.^12^

The models were done in Stata using the *fmm* command.^13^ We fitted a mixture of two linear regression models for the optical density for the two serologic statuses.

#### Visual inflection point

First, the optical density was sorted from the lowest to the highest. Then the point where the curvature of the graph changes (from ‘predominantly horizontal to predominantly vertical’) was identified visually by eyeballing.^12,15^ We purposively selected 10 respondents with a range of backgrounds and career levels to identify the point. The mean of these values was taken as the cutoff.

#### Change point analysis

A change point is a point where there is a sudden change in the statistical characteristics in the observed sequence.^16,17^ We used a Bayesian Poisson model using random-walk Metropolis-Hastings sampling to identify the change point in Stata using the *bayesmh* command.^18^ We used a uniform prior on the values corresponding to the mean minus one standard deviation and mean plus one standard deviation (mean-1SD and mean+1SD) of the resulting visual inflection point cutoffs. For the initial change point parameter values, we used the mean from visual inflection point results.

## Results

There were two samples from Jambi with missing age data; these samples were not included in the data analysis. A total of 1033 samples were included in the data analysis (Supplementary Data 1, Supplementary Table S3). Accounting for all variables, there were 40 participants with incomplete observations. The complete dataset can be viewed in Supplementary Data 2.

### Participants’ characteristics

More than half (n=538/1033, 52.1%) of the included samples were from male participants (Supplementary Data 1, Supplementary Table S3). Most of the participants were in the 16–30 age brackets (31.5%). Over 9 in 10 participants (n=990, 95.8%) had a history of fever. The median platelet level was low.

### Cutoff selection

Table 1 shows the outcome of the finite mixture models of IgM optical density values. The mean of the lower class, i.e. modelled seronegative subpopulation, was 0.15 (95% CI: 0.14 to 0.17). The cutoff for IgM was 0.4891≈0.49. Table 1 showed the outcome of the finite mixture models of IgG optical density values. The mean of the lower class, i.e. modelled seronegative subpopulation, was 0.03 (95% CI: 0.03 to 0.04). The cutoff for IgG was 0.1257≈0.13. The differences between IgM and IgG-positive cutoff calculated using FMMs and other methods were less than 0.02 and 0.1, respectively (Table 2).

**Table 1.** Finite mixture models outcome

		95% Confidence interval		
	Mean	Lower limit	Upper limit	Variance
IgM				
Lower class	0.15	0.14	0.17	0.01
Upper class	0.25	0.21	0.29	0.08
IgG				
Lower class	0.035	0.03	0.04	0.001
Upper class	0.19	0.12	0.25	0.06

**Table 2.** The comparison of cutoffs from three methods

	Visual inflection point	Change point analysis	Finite mixture models
IgM cutoff	0.47	0.47	0.49
IgG cutoff	0.18	0.22	0.13
IgM seropositivity	57 (5.5%)	57 (5.5%)	47 (4.6%)
IgG seropositivity	27 (2.6%)	21 (2.0%)	45 (4.4%)
Overall seropositivity*	83 (8.0%)	78 (7.6%)	91 (8.8%)

*The proportion of samples with positive IgM and/or IgG against scrub typhus.

### Optical density values distribution

#### IgM

**Figure 1.**
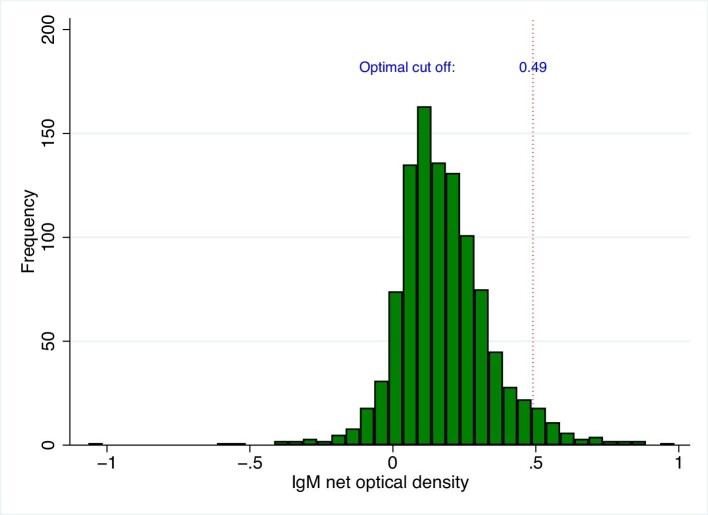
The histogram of IgM data. The red dotted line represents the 0.49 cutoff.

The IgM values ranged from −1.07 to 0.97, with mean (SD) of 0.18 (0.17) and median (Q1–Q3) of 0.16 (0.07–0.27) (Figure 1).

#### IgG

**Figure 2.**
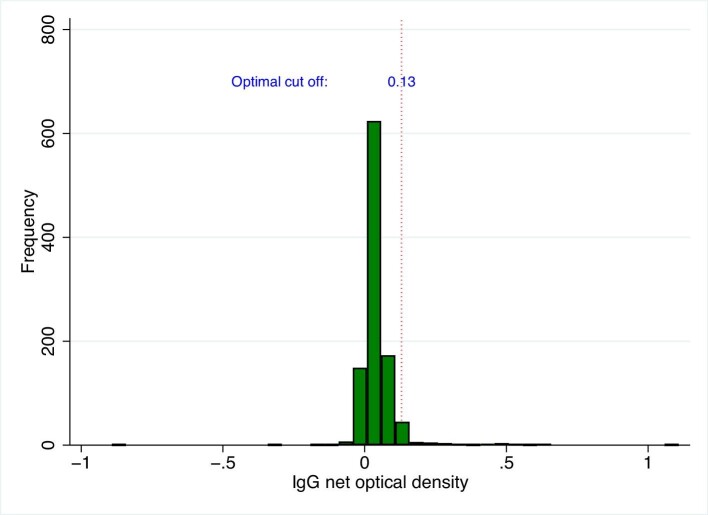
The histogram of IgG data. The red dotted line represents the 0.13 cutoff.

The IgG values ranged from −0.89 to 1.09, with mean (SD) of 0.05 (0.08) and median (Q1–Q3) of 0.03 (0.02–0.06) (Figure 2).

### Main results

The overall seropositivity, i.e. when IgM and/or IgG against scrub typhus was positive, was 8.8% (n=91/1033). The number of IgM-positive samples was higher than the IgG-positive samples: 47 (4.6%) and 45 (4.4%) for IgM and IgG, respectively. There was one sample with positive IgM and IgG.

The number and proportion of overall seropositivity was highest in Jambi (n=34/350, 9.7%) (Table 3). The IgM seropositivity was highest in Jambi as well (n=20/350, 5.7%). For IgG seropositivity, all the sites had 15 positive samples each, but the proportion was highest in Tabanan (n=15/268, 5.6%). There was no association between the overall positivity status and sample collection sites (p-value = 0.69).

**Table 3.** Seropositivity proportion of each site

	Jambi	Denpasar	Tabanan	Overall
IgM positive	20 (5.7%, 95% CI: 3.7 to 8.7%)	18 (4.3%, 95% CI: 2.8 to 6.8%)	9 (3.4%, 95% CI: 1.8 to 6.3%)	47 (4.6%, 95% CI: 3.4 to 6.0%)
IgG positive	15 (4.3%, 95% CI: 2.6 to 7.0%)	15 (3.61%, 95% CI: 2.2 to 5.9%)	15 (5.6%, 95% CI: 3.4 to 9.0%)	45 (4.4%, 95% CI: 3.3 to 5. 8%)
Overall positive*	34 (9.7%, 95% CI: 7.0 to 13.3%)	33 (8.0%, 95% CI: 5.7 to 11.0%)	24 (9.0%, 95% CI: 6.1 to 13.0%)	91 (8.8%, 95% CI: 7.2 to 10.7%)

*The proportion of samples with positive IgM and/or IgG against scrub typhus.

95% CI calculated using Wilson score interval.

There were 7.4% (n=40/538) males and 10.3% (n=51/495) females with overall positive status. There was no statistically significant difference between the two proportions (p-value = 0.10, 95% CI: −0.06 to 0.01). With one year increase in age, the odds of having overall positive status are increased by 1% (p-value = 0.03, 95% CI: 1.00 to 1.02).

There was a statistically significant difference in IgM values between males and females (p-value = 4.65×10^−5^, 95% CI: −0.06 to −0.02), suggesting lower IgM values in males. The difference between the two groups for the mean of IgG values was not statistically significant (p-value = 0.54, 95% CI: −0.01 to 0.01).

There was a negative correlation between the IgM values and age (rho = −0.07, p-value = 0.02). The IgM optical density, on average, decreases by 0.0009 for every one-year increase in age (p-value = 0.003) (Table 4). Only 0.9% of the variation in IgM values can be explained by the model containing only age. There was a positive correlation between the IgG values and age (rho = 0.23, p-value = 2.92×10^−14^). Simple linear regression showed a significant relationship between age and IgG values (p-value = 1.61×10^−10^). The IgG optical density, on average, increases by 0.0009 for every one-year increase in age (Table 4). Only 3.9% of the variation in IgG values can be explained by the model containing only age.

**Table 4.** Linear regression summary for age predicting IgM and IgG value

	Estimate	95% CI	p-Value
IgM			
Intercept	0.20	0.18 to 0.22	<2×10^−16^
Age	−0.0009	−0.002 to −0.0003	0.003
IgG			
Intercept	0.02	0.01 to 0.03	8.70×10^−8^
Age	0.0009	0.0006 to 0.001	1.61×10^−10^

For IgM values: R-squared: 0.0083, adjusted R-squared: 0.0074. For IgG values: R-squared: 0.0389, adjusted R-squared: 0.0380.

The proportion of dengue negative samples within the overall seropositive and IgM positive scrub typhus sample were 79.1% (n=72/91) and 3.8% (n=39/1033), respectively (Supplementary Data 1, Supplementary Table S4). There was no statistically significant association between overall scrub typhus seropositivity and dengue infection (p-value = 0.19, 95% CI: −0.02 to 0.08).

## Discussion

We used in-house optimized and validated ELISA to test for IgM and IgG against scrub typhus to analyse 1033 archived serum samples from acute febrile illness studies in Indonesia. We used FMMs to determine cutoffs. The cutoffs used for IgM and IgG were 0.49 and 0.13, respectively. The IgM seropositivity across all sites was 4.6% (95% CI: 3.4 to 6.0%), while the IgG seropositivity was 4.4% (95% CI: 3.3 to 5.8%). The overall seropositivity was 8.8% (95% CI: 8.1 to 11.7%).

Seropositive status in this study would indicate that there was exposure to *O. tsutsugamushi*. Positivity might also indicate acute infection, particularly in the dengue negative and scrub typhus IgM positive group. However, that remains uncertain because we did not have convalescent samples and gold standard testing. IgM against scrub typhus seems to peak early in the infection (5.2 days) and wanes rapidly.^19,20^ Using data from Thailand and India, Aiemjoy et al. and Schmidt et al. have shown that after 5.3 months^19^ and 2.7 months^20^ 50% of participants had IgM OD of <1, respectively. Scrub typhus ELISA might return a positive result due to a cross reaction with antibodies generated by other febrile illnesses.^21^ If we want to ensure few false positives, we could increase the cutoff to diagnose an acute case. However, we think the cutoff used here is already specific, as it was calculated by adding 3SD to the mean of the assumed negative distribution, instead of 1SD or 2SD. For acute samples, other diagnostic methods could be more ideal given the timing, i.e. PCR or other nucleic acid detection-based methods. However, since the overarching aim of this study was to understand the distribution of scrub typhus in Indonesia, the use of serology method was justified.

The seropositive proportion might not reflect the seroprevalence of scrub typhus in the community. Since the samples were from acute febrile participants consecutively recruited in hospitals, it might select people who were febrile due to scrub typhus, thereby increasing the proportion.

The use of febrile study samples might explain the findings in this survey. In the case of dengue, seroprevalence studies in non-febrile participants, e.g. blood donors, in endemic locations usually showed higher IgG compared to IgM seropositivity.^22–24^ In this study, the IgM was higher than the IgG seropositivity. Since the samples were collected in participants with acute fever, this suggests that scrub typhus was the cause of fever in some of these febrile cases. We only found one participant with both positive IgM and IgG. This could be caused by the cutoffs used. Here, we used a more specific cutoff to minimize false positives in concordance with our aim to define the distribution and frequency of exposure to scrub typhus. The sampling methods used by the acute febrile illness studies would also influence the antibody dynamics detected here. These results could be found in an area with low force of infection, where the participants with positive IgM would represent primary infections and therefore, there would be no IgG positivity. The IgG positive group would most likely be those who have had previous exposure or infections but were not acutely infected at the time of sampling.

The proportion of IgG seropositivity found here was slightly higher than in one recently published study from Indonesia (4.4% vs 3.8%).^25^ This could be caused by the wider eligibility criteria applied in the previous study, i.e. no exclusion on people presenting with focal signs/symptoms, e.g. gastrointestinal or respiratory symptoms, or difference in cutoffs. The previous study collected samples from tertiary referral hospitals,^25^ while this study population also included a district hospital in semi-urban area.

Many previous studies used 0.5 as the optical density cutoff.^26^ Here, we used 0.49 for IgM and 0.13 for IgG. We performed three methods to calculate the cutoff and they largely agree. However, this cutoff might not be suitable for other sites or populations as they may have different background immunity.

The visual inflection point, change point analysis and finite mixture modelling can be used to determine the cutoff when there is no sample from an unexposed population or when the gold standard diagnostic method is unavailable. The visual inflection point method is simple and does not need any calculations, models or software. In this project, the mean of the respondents’ answer is quite close to the results from other methods. However, if we look at individual responses (Supplementary Data 1, Supplementary Table S5), some are quite far from the mean. Ensuring there are enough respondents could be important when using this technique. Here, with 10 respondents, the resulting mean for IgM and IgG was similar with the other methods. It is also important to clearly define inflection point to the respondents.

We also performed a change point analysis using Bayesian approach. It is important to ensure that we input appropriate and specific priors, since the results of this method can change according to which priors were used.

To determine the cutoffs using FMMs, we need to define one number in the command, i.e. the number of subpopulations. Knowledge of the disease is essential to be able to define this number appropriately. Compared to the change point analysis this method could be less complicated. The results are also more intuitive to interpret.

We can conclude that there has been exposure to scrub typhus at all sites, and although some patients could be ill with acute scrub typhus, most of those who were IgG seropositive probably had previous exposure to *Orientia tsutsugamushi* and scrub typhus was not the cause of their acute illness. Jambi and Denpasar being provincial capitals (urban areas) might partly explain this. Although not a major cause of acute fever, keeping in mind that there was active transmission, it is still important to have a low threshold of suspicion of the disease—especially when there are cases of acute fever that are dengue-negative and do not respond to beta-lactam antibiotic treatment. This study also provided first scrub typhus data for Tabanan, contributing to the understanding of scrub typhus in Indonesia.

The use of archived sera may have optimized the value of the samples. However, the primary study was not specifically designed for the purpose of the current study and there might be some antibody degradation during storage. Due to the limited resources and the COVID-19 pandemic making primary clinical research difficult, we chose to use these archived samples.

In the future, seroprevalence surveys in the community and inclusion of scrub typhus in the diagnostic panel of acute febrile illness studies in more areas (particularly rural areas) would be crucial in understanding scrub typhus distribution in Indonesia. Serosurveys in asymptomatic individuals would also help in determining background immunity and thus cutoffs. Exploring factors associated with exposure to scrub typhus is also important. Accurate and accessible point-of-care tests would be invaluable in supporting future scrub typhus studies.

## Conclusions

*O. tsutsugamushi* exposure in humans occurs at all sites analysed. Though scrub typhus might not be the most common cause of acute fever in Jambi, Denpasar and Tabanan, it is still important to consider in cases not responding to beta-lactam antibiotics. We still need further studies in other regions of Indonesia, particularly in rural areas.

## Supplementary Material

trad094_Supplemental_Files

## Data Availability

The data underlying this article are available in the article and in its online supplementary material.
