# Supplementary material for: Scrub typhus in Indonesia: A cross-sectional analysis of archived fever studies samples
Source: Trans R Soc Trop Med Hyg. 2024 Jan 11;118(5):321–7. doi: 10.1093/trstmh/trad094 (PMC11062201; doi:10.1093/trstmh/trad094)
Supplement: trad094_Supplemental_Files [file trad094_supplemental_files.zip › Supplementary Data 1.docx]

Table S1. The characteristics of each AFI study

| Sites | Jambi, Sumatra^9^ | Denpasar, Bali^10^ | Tabanan, Bali |
| --- | --- | --- | --- |
| Sample collection time | December 2014–December 2015 | January 2017–July 2018 | January 2017–July 2018 |
| Settings | Urban | Urban | Semi-urban |
| Hospital | Private hospital | Public district hospital | Public district hospital |
| Inclusion criteria | Patients presenting within 5 days of onset with fever > 38°C and at least one of dengue-like clinical manifestations including retro-orbital pain, joint pain, malaise, and rash. | Patients 14 years and older presenting with fever > 38°C and at least one of dengue-like clinical manifestations including retro-orbital pain, joint pain, malaise, and rash. | Patients ≥ 6 months presenting with fever (oral temperature ≥ 38°C and/or history of fever) for < 7 days and leukocyte count ≤ 10,000 |
| Exclusion criteria | None | History of chronic diseases including diabetes mellitus, human immunodeficiency syndrome, chronic kidney, liver, lung, and heart disease. | Illness is considered as respiratory or gastrointestinal infection. |
| Number of samples | 352 | 415 | 268 |

Table S2. STROBE Statement—Checklist of items that should be included in reports of ***cross-sectional studies***

|  | Item No | Recommendation | Part |
| --- | --- | --- | --- |
| **Title and abstract** | 1 | (*a*) Indicate the study’s design with a commonly used term in the title or the abstract | Title page and abstract |
|  |  | (*b*) Provide in the abstract an informative and balanced summary of what was done and what was found | Abstract |
| Introduction | | | |
| Background/rationale | 2 | Explain the scientific background and rationale for the investigation being reported | Introduction par. 2 & 3 |
| Objectives | 3 | State specific objectives, including any prespecified hypotheses | Introduction par. 4 |
| Methods | | | |
| Study design | 4 | Present key elements of study design early in the paper | Methods par. 1 |
| Setting | 5 | Describe the setting, locations, and relevant dates, including periods of recruitment, exposure, follow-up, and data collection | Methods par. 2, Table 1 |
| Participants | 6 | (*a*) Give the eligibility criteria, and the sources and methods of selection of participants | Methods par. 2, Table S1 |
| Variables | 7 | Clearly define all outcomes, exposures, predictors, potential confounders, and effect modifiers. Give diagnostic criteria, if applicable | Methods par. 5 |
| Data sources/ measurement | 8* | For each variable of interest, give sources of data and details of methods of assessment (measurement). Describe comparability of assessment methods if there is more than one group | Methods par. 5 & 6 |
| Bias | 9 | Describe any efforts to address potential sources of bias | Not applicable since data and samples were from previous studies. This is discussed in Discussion par. 3 & 4. |
| Study size | 10 | Explain how the study size was arrived at | Methods par. 3 |
| Quantitative variables | 11 | Explain how quantitative variables were handled in the analyses. If applicable, describe which groupings were chosen and why | Methods par. 5 |
| Statistical methods | 12 | (*a*) Describe all statistical methods, including those used to control for confounding | Methods par. 5 |
|  |  | (*b*) Describe any methods used to examine subgroups and interactions | Methods par. 5 |
|  |  | (*c*) Explain how missing data were addressed | Methods par. 5 |
|  |  | (*d*) If applicable, describe analytical methods taking account of sampling strategy | N/A |
|  |  | (*e*) Describe any sensitivity analyses | N/A |
| Results | | | |
| Participants | 13* | (a) Report numbers of individuals at each stage of study—eg numbers potentially eligible, examined for eligibility, confirmed eligible, included in the study, completing follow-up, and analysed | N/A |
|  |  | (b) Give reasons for non-participation at each stage | N/A |
|  |  | (c) Consider use of a flow diagram | N/A |
| Descriptive data | 14* | (a) Give characteristics of study participants (eg demographic, clinical, social) and information on exposures and potential confounders | Results par. 2, Table S3 |
|  |  | (b) Indicate number of participants with missing data for each variable of interest | Results par. 1 |
| Outcome data | 15* | Report numbers of outcome events or summary measures | Results par. 1 |
| Main results | 16 | (*a*) Give unadjusted estimates and, if applicable, confounder-adjusted estimates and their precision (eg, 95% confidence interval). Make clear which confounders were adjusted for and why they were included | Results par. 8 |
|  |  | (*b*) Report category boundaries when continuous variables were categorized | N/A |
|  |  | (*c*) If relevant, consider translating estimates of relative risk into absolute risk for a meaningful time period | N/A |
| Other analyses | 17 | Report other analyses done—eg analyses of subgroups and interactions, and sensitivity analyses | N/A |
| Discussion | | | |
| Key results | 18 | Summarise key results with reference to study objectives | Discussion par. 1 |
| Limitations | 19 | Discuss limitations of the study, taking into account sources of potential bias or imprecision. Discuss both direction and magnitude of any potential bias | Discussion par. 11 |
| Interpretation | 20 | Give a cautious overall interpretation of results considering objectives, limitations, multiplicity of analyses, results from similar studies, and other relevant evidence | Discussion par. 13 |
| Generalisability | 21 | Discuss the generalisability (external validity) of the study results | Discussion par. 5 & 6 |
| Other information | | | |
| Funding | 22 | Give the source of funding and the role of the funders for the present study and, if applicable, for the original study on which the present article is based | Funding |

*Give information separately for exposed and unexposed groups.

**Note:** An Explanation and Elaboration article discusses each checklist item and gives methodological background and published examples of transparent reporting. The STROBE checklist is best used in conjunction with this article (freely available on the Web sites of PLoS Medicine at http://www.plosmedicine.org/, Annals of Internal Medicine at http://www.annals.org/, and Epidemiology at http://www.epidem.com/). Information on the STROBE Initiative is available at www.strobe-statement.org.

Table S3. Participants' characteristics

| **Characteristics** | **Jambi** | **Denpasar** | **Tabanan** | **Overall** |
| --- | --- | --- | --- | --- |
| **Sex** | | | | |
| Male – n (%) | 177 (50.6%) | 207 (49.9%) | 154 (57.5%) | 538 (52.1%) |
| Female – n (%) | 173 (49.4%) | 208 (50.1%) | 114 (42.5%) | 495 (47.9%) |
| **Age** | | | | |
| 0–5 – n (%) | 53 (15.1%) | 66 (15.9%) | 43 (16.0%) | 162 (15.7%) |
| 6–15 – n (%) | 72 (20.6%) | 81 (19.5%) | 48 (17.9%) | 201 (19.5%) |
| 16–30 – n (%) | 115 (32.9%) | 135 (32.5%) | 75 (28.0%) | 325 (31.5%) |
| 31–45 – n (%) | 76 (21.7%) | 76 (18.3%) | 44 (16.4%) | 196 (19.0%) |
| 46–60 – n (%) | 27 (7.7%) | 39 (9.4%) | 43 (16.0%) | 109 (10.6%) |
| >60 – n (%) | 7 (2.0%) | 18 (4.3%) | 15 (5.6%) | 40 (3.9%) |
| **Clinical manifestations** | | | | |
| Fever – n (%) | 332 (94.9%) | 402 (96.9%) | 256 (95.5%) | 990 (95.9%) |
| Malaise – n (%) | 300 (85.7%) | 304 (73.3%) | 102 (38.1%) | 706 (68.3%) |
| Nausea and/or vomiting – n (%) | 239 (68.3%) | 309 (74.5%) | 147 (54.9%) | 695 (67.3%) |
| Headache – n (%) | 248 (70.9%) | 227 (54.7%) | 160 (59.7%) | 635 (61.5%) |
| Loss of appetite – n (%) | 138 (39.4%) | 250 (60.2%) | 74 (27.6%) | 462 (44.7%) |
| Myalgia – n (%) | 125 (35.2%) | 127 (30.6%) | 82 (30.6%) | 334 (32.3%) |
| Abdominal pain – n (%) | 187 (53.4%) | 40 (9.6%) | 21 (7.8%) | 248 (24.0%) |
| Arthralgia – n (%) | 41 (11.7%) | 132 (31.8%) | 58 (21.6%) | 231 (22.4%) |
| Retroorbital pain – n (%) | 15 (4.3%) | 49 (11.8%) | 31 (11.6%) | 95 (9.2%) |
| Rash – n (%) | 47 (13.4%) | 27 (6.5%) | 9 (3.4%) | 83 (8.0%) |
| Bleeding – n (%) | 2 (0.6%) | 42 (10.1%) | 6 (2.2%) | 50 (4.8%) |
| Dyspnoea – n (%) | 6 (1.7%) | 17 (4.1%) | 2 (0.8%) | 25 (2.4%) |
| Altered consciousness – n (%) | 1 (0.3%) | 0 (0.0%) | 1 (0.4%) | 2 (0.2%) |
| **Laboratory parameters** | | | | |
| Haemoglobin – median (Q1 – Q3) | 13 (11.90 – 14.10) | 13.1 (11.80 – 14.10) | 14.3 (13.11 – 15.70) | 13.3 (12.10 – 14.68) |
| Haematocrit – median (Q1 – Q3) | 38 (34.90 – 40.70) | 37.9 (34.30 – 41.60) | 42.8 (39.35 – 47.45) | 43.8 (37.8 – 184.00) |
| Platelet – median (Q1 – Q3) | 228 (181.75 – 281.00) | 163 (118.50 – 239.00) | 189 (154.00 – 230.50) | 127 (40.08 – 199.00) |
| Leukocyte – median (Q1 – Q3) | 7.67 (5.28 – 10.47) | 5.32 (3.60 – 7.08) | 6.16 (4.68 – 7.82) | 6.25 (4.39 – 8.41) |
| **Total** | 350 | 415 | 268 | 1033 |

Table S4. The scrub typhus overall seropositivity status and dengue infection

|  | **Dengue positive** | **Dengue negative** | **Total** |
| --- | --- | --- | --- |
| Scrub typhus overall seropositive | 19 | 72 | 91 |
| - Scrub typhus IgM positive* | 8 | 39 | 47 |
| - Scrub typhus IgG positive* | 11 | 34 | 45 |
| Scrub typhus overall seronegative | 147 | 795 | 932 |
| Total | 166 | 867 | 1,033 |

*Numbers not counted again in totals

Table S5. The visual inflection points identified by the respondents

| **Respondent** | **IgM OD cut-off** | **IgG OD cut-off** |
| --- | --- | --- |
| 1 | 0.45 | 0.18 |
| 2 | 0.40 | 0.20 |
| 3 | 0.57 | 0.17 |
| 4 | 0.30 | 0.10 |
| 5 | 0.47 | 0.18 |
| 6 | 0.50 | 0.15 |
| 7 | 0.55 | 0.18 |
| 8 | 0.50 | 0.30 |
| 9 | 0.60 | 0.20 |
| 10 | 0.40 | 0.18 |
| Mean | 0.47 | 0.18 |
| SD | 0.09 | 0.05 |
